# Fully end-to-end deep-learning-based diagnosis of pancreatic tumors

**DOI:** 10.7150/thno.52508

**Published:** 2021-01-01

**Authors:** Ke Si, Ying Xue, Xiazhen Yu, Xinpei Zhu, Qinghai Li, Wei Gong, Tingbo Liang, Shumin Duan

**Affiliations:** 1 Department of Hepatobiliary and Pancreatic Surgery of the First Affiliated Hospital, State Key Laboratory of Modern Optical Instrumentation, Zhejiang University School of Medicine, Hangzhou 310003, China.; 2 College of Optical Science and Engineering, Zhejiang University, Hangzhou 310027, China.; 3 Research Units for Emotion and Emotion Disorders, Chinese Academy of Medical Sciences, MOE Frontier Science Center for Brain Research and Brain-Machine Integration, School of Brain Science and Brain Medicine, Zhejiang University, Hangzhou 310058, China.; 4 Department of Radiology of the Second Affiliated Hospital, Zhejiang University School of Medicine, Hangzhou 310009, China.; 5 Zhejiang Provincial Key Laboratory of Pancreatic Disease, the First Affiliated Hospital, Zhejiang University School of Medicine, Hangzhou, China.; 6 Zhejiang University Cancer Center, Hangzhou, China.

**Keywords:** artificial intelligence (AI), computed tomography (CT), deep learning, convolutional neural network (CNN), tumor

## Abstract

Artificial intelligence can facilitate clinical decision making by considering massive amounts of medical imaging data. Various algorithms have been implemented for different clinical applications. Accurate diagnosis and treatment require reliable and interpretable data. For pancreatic tumor diagnosis, only 58.5% of images from the First Affiliated Hospital and the Second Affiliated Hospital, Zhejiang University School of Medicine are used, increasing labor and time costs to manually filter out images not directly used by the diagnostic model.

**Methods:** This study used a training dataset of 143,945 dynamic contrast-enhanced CT images of the abdomen from 319 patients. The proposed model contained four stages: image screening, pancreas location, pancreas segmentation, and pancreatic tumor diagnosis.

**Results:** We established a fully end-to-end deep-learning model for diagnosing pancreatic tumors and proposing treatment. The model considers original abdominal CT images without any manual preprocessing. Our artificial-intelligence-based system achieved an area under the curve of 0.871 and a F1 score of 88.5% using an independent testing dataset containing 107,036 clinical CT images from 347 patients. The average accuracy for all tumor types was 82.7%, and the independent accuracies of identifying intraductal papillary mucinous neoplasm and pancreatic ductal adenocarcinoma were 100% and 87.6%, respectively. The average test time per patient was 18.6 s, compared with at least 8 min for manual reviewing. Furthermore, the model provided a transparent and interpretable diagnosis by producing saliency maps highlighting the regions relevant to its decision.

**Conclusions:** The proposed model can potentially deliver efficient and accurate preoperative diagnoses that could aid the surgical management of pancreatic tumor.

## Introduction

Pancreatic cancer, one of the most frequent, has poor prognosis and is usually fatal 1-3. Patients without tumor need only further observation, while a pancreatic tumor diagnosis requires urgent action and a definite surgical plan. The risk of exacerbation and death increases if treatment is delayed, making the accurate diagnosis of pancreatic tumor crucial to its successful surgical treatment.

Artificial intelligence (AI) can help improve the accuracy of image interpretation and make diagnostic expertise more widely available 4, 5. However, AI methods for diagnosing pancreatic tumors are not well developed as the task is especially challenging. First, the target is highly variable in shape, size, and location and occupies only a very small fraction of the entire CT image. The pancreas occupies only about 1.3% of each CT image in our CT dataset. The remaining information are from other organs such as the liver, stomach, intestines and image background, which barely impact the diagnosis by the AI model. Furthermore, a tumor's high similarity to the surrounding tissues further reduces accuracy and diagnostic efficiency 6. A third point is the lack of a suitable pancreas image dataset, which directly affects the development of an AI model.

Previous studies have attempted to solve these problems. An effective method is pancreas segmentation. Chakraborty *et al*. predicted high-risk intraductal papillary mucinous neoplasms (IPMN) of the pancreas based on random forests and support vector machine learning applied to manually segmented CT images 7. Wei *et al*. presented a support vector machine system containing 24 guideline-based features and 385 radiomics high-throughput features combined with regions of interest (ROI) marked by a radiologist to diagnose pancreatic serous cystic neoplasms (SCN) 8. With the development of deep-learning frameworks 9, researchers have been able to construct effective deep encoder-decoder networks 10 for pancreas segmentation, boosting diagnostic accuracy 11-14. Zhu *et al.* reported a multi-scale segmentation method for screening pancreatic ductal adenocarcinoma (PDAC) by checking if a sufficient number of voxels were segmented as tumors 15. Liu *et al.* segmented the pancreas first, and then classified abnormalities to detect PDAC 16. However, efficiently obtaining an immediate diagnosis and treatment recommendation without increasing medical specialists' workload or procedural costs is a major problem. As the original patient data (taken from hospital records) contains CT examination diagnosis reports and images from different imaging planes and angiography phases, the proportion of effective CT images that can be used for diagnosis is small. Therefore, the key to successful clinical application of a deep-learning framework is detailed automated preprocessing of the original data.

This study proposes a fully end-to-end deep-learning (FEE-DL) model for the automatic diagnosis of pancreatic tumors from original abdominal CT images. The model's methodology has four steps for locating pancreatic tumors from the original data: image screening, pancreas location, pancreas segmentation, and pancreatic tumor diagnosis. The model was trained with a dataset of 143,945 clinical CT images from 319 patients, and tested on 107,036 clinical CT images from 347 patients. Its quick and accurate pancreatic tumor diagnoses can potentially aid surgical decision making.

## Methods

### Dataset preparation

Dynamic contrast-enhanced CT images of the abdomen were utilized by the FEE-DL model. The study was approved by the hospital's Institutional Review Board and informed consent was obtained from patients and healthy control subjects. The training dataset were randomly collected from the Second Affiliated Hospital, Zhejiang University School of Medicine (Zhejiang, China). The independent testing dataset were randomly collected from both the First Affiliated Hospital and the Second Affiliated Hospital, Zhejiang University School of Medicine (Zhejiang, China). All patients underwent preoperative abdominal contrast-enhanced CT scanning. Both hospitals used two different scanners: the first used a 64-slice and a 256-slice CT (Philips Healthcare), and the second used a 40-slice new dual source CT (Siemens AG) and a 320-slice CT (Toshiba Medical Systems). Both institutions used the same CT scanning parameters: 120 kVp tube voltage, 125-300 mAs tube current, 0.6-1.25 mm pitch, 3-5 mm slice thickness, and 3-5 mm reconstruction interval. Experienced radiologists labeled the location of the pancreas in the arterial phase of CT scans using Adobe PhotoShop software and classified the patients as either with or without pancreatic tumor.

Model training using a dataset of 143,945 clinical, 512 × 512 pixel, 8-bit, CT images: 133,591 from 284 patients with tumors and 10,354 from 35 tumor-free control patients. Overall, there were 211 males and 108 females (age range 37-90 years, mean 63.3 years).

**Table 1** lists the types of pancreatic tumor and their frequency in the training and testing datasets. Pancreatic cancer (PDAC) and pancreatic tumors such as IPMN, pancreatic neuroendocrine tumors (PNET), SCN, and 'Other' are considered as positive cases. Rare cases or lesions on the pancreas caused by adjacent abdominal organ diseases are labeled as 'Other', including gallbladder cancer, cholangiocarcinoma, ampullary carcinoma, duodenal carcinoma and metastatic cancer. To effectively enlarge our training dataset to improve the robustness of the proposed model, we performed four data augmentation operations: randomly setting brightness in range (-0.8, 0.8), randomly setting contrast in the range (-0.8, 0.8), random elastic transformation with an alpha affine of 30, and random cropping to 450 × 450 pixels followed by resizing to 512 × 512 pixels. The model was then tested with an independent dataset of 90,194 images from 265 tumor patients and 16,842 images from 82 control patients (216 males and 131 females, age range 24-88 years, mean 61.8 years). **Table 2** lists detailed patient characteristics.

### Image screening

**Figure 1:**
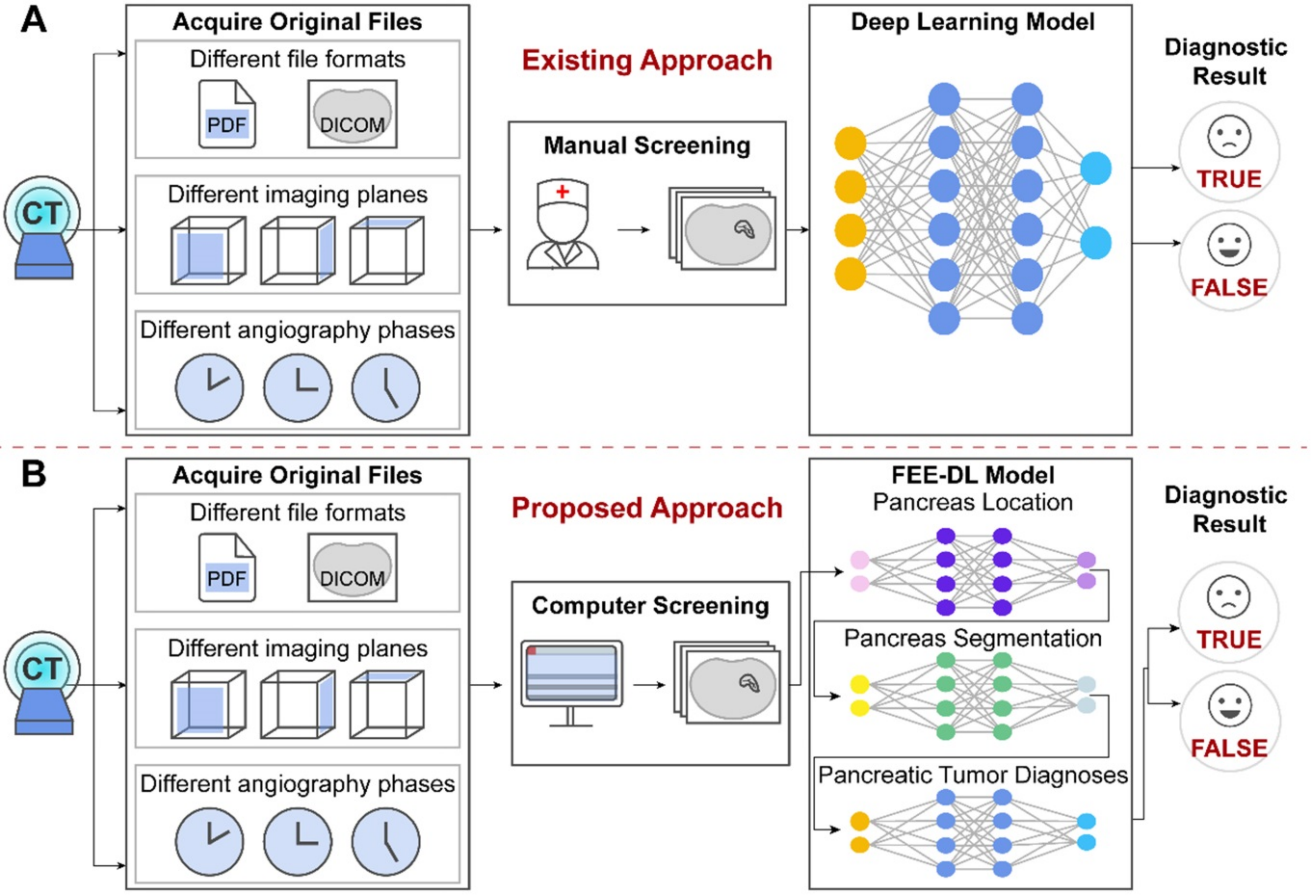
The original files obtained from the hospitals contain different file formats, different imaging planes and different angiography phases. **(A)** Artificial intelligence approaches currently used for pancreatic diagnosis focus on the analysis of valid CT images, and ignore the importance of screening the original data at an early stage. **(B)** Our proposed FEE-DL model first screens out transverse plane CT images containing the pancreas from complex original files before deep-learning diagnosis.

The original patient data obtained from the hospitals cover multiple files. However, existing methods focus on the analysis of CT images containing the pancreas, and ignore the importance of screening the original data at an early stage, as shown in **Figure 1A**. The proposed model first screens out transverse plane CT images containing the pancreas before deep-learning diagnosis **(Figure 1B)**.

**Figure 2:**
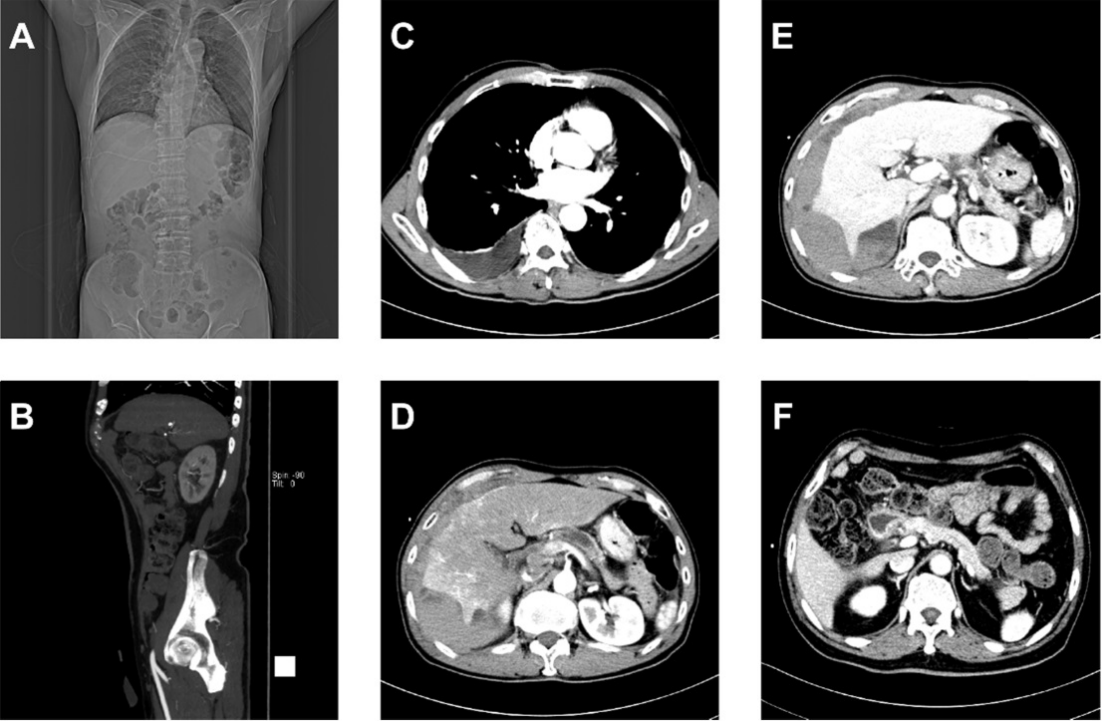
Multiplex original clinical data. **(A-C)** Images not directly used by the FEE-DL model containing **(A)** coronal plane CT scan, **(B)** sagittal plane CT scan, and **(C)** CT scan without pancreas. **(D)** Arterial, **(E)** venous, and **(F)** delayed phase CT scans.

**Figure 2** shows that the dataset we established is complex with three important characteristics: text reports (CT examination diagnosis reports and patient protocols), different imaging planes (coronal, sagittal, and transverse), and different angiography phases (arterial, venous, and delayed or portal vein phase). To control the image quality, screening selects only transverse plane CT images containing the pancreas.

Each image in the dataset contains attributes such as 'Patient Name', 'Image shape', and 'Series Description'. The model screens images according to 'Image shape' being 512 × 512 and 'Series Description' being 'Arterial phase', 'Venous phase', or 'Delayed phase'. In consideration of the different specifications of scanners, we enhanced the contrast of the images and then normalized them to 0-255 to highlight the pancreas structure and increase the versatility of the FEE-DL model.

### Model establishment

**Figure 3:**
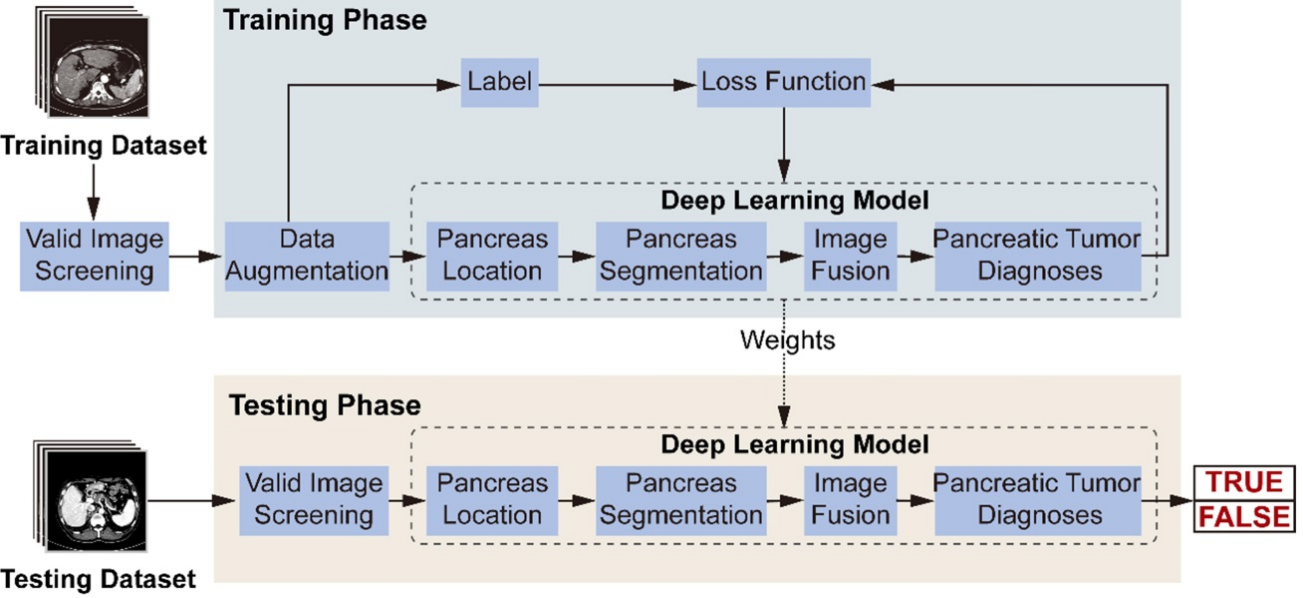
Workflow diagram of the model's training and testing phase. In the training phase, after valid images screening and data augmentation from the original abdominal CT images, we constructed a deep-learning model involving pancreas location, pancreas segmentation, image fusion and pancreatic tumor diagnoses. The loss function is calculated according to the prediction and label, and the weights of the neural networks are updated according to the back-propagation algorithm. The best weights are fixed for subsequent use on the testing dataset to diagnose pancreatic tumor.

**Figure 3** illustrates the model's workflow. In the training phase, we screened valid images for diagnosis from the original abdominal CT images. After data augmentation, we constructed a deep-learning model involving three connected sub-networks, which are widely used in medical image recognition with demonstrated efficiency 17, 18. ResNet18 is used to recognize images containing the pancreas. **Figure 2C** shows transverse plane CT images without the pancreas, and are thus not directly used for the FEE-DL model diagnosis. U-Net32 makes predictions on each image pixel, yielding binary results for pancreas segmentation. During subsequent image fusion, we added texture features of the pancreas to the segmented result to provide a richer diagnosis basis for the next sub-network. As the last neural network in the FEE-DL model, ResNet34 is used to diagnose the presence of pancreatic tumor. The loss function is calculated as the deviation between the output of the neural network and the label, and the weights of each layer are updated according to the back-propagation algorithm. We determined the best weights with the minimal loss value, and fixed them for subsequent use on the testing dataset.

### Architectures of deep neural networks

**Figure 4:**
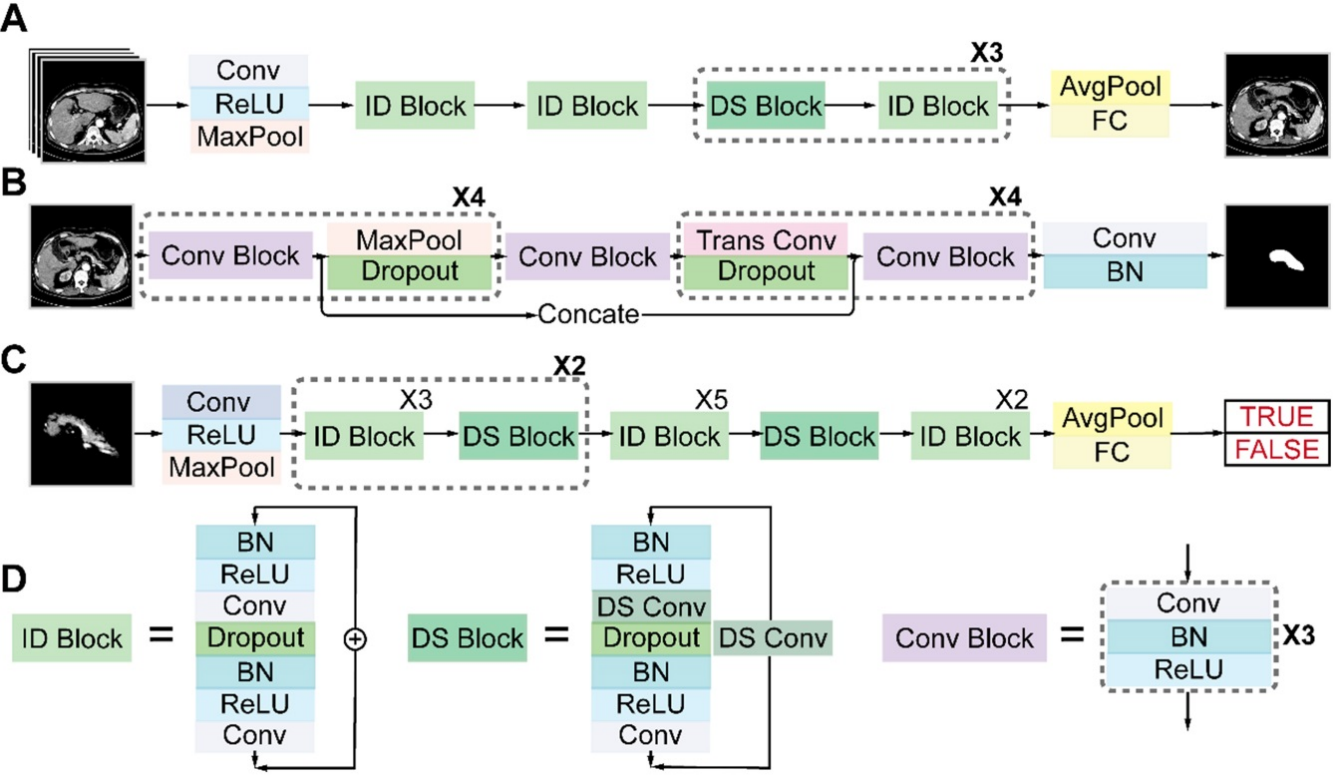
Architectures of the three sub-networks: **(A)** ResNet18 for pancreas location, **(B)** U-Net32 for pancreas segmentation, and **(C)** ResNet34 for pancreatic tumor diagnosis. **(D)** Detailed structures of the identity (ID), down sampling (DS), and convolution (Conv) blocks. (AvgPool, average-pooling; BN, batch normalization; Concate, concatenation; FC, fully connected; MaxPool, max-pooling; ReLU, rectified linear unit; Trans, transposed).

**Figure 4** shows the detailed architectures of the three sub-networks (see also **Table S1-S3**). ResNet18 and ResNet34 have similar architectures but different depths, as shown in **Figures 4A** and **4C**. The convolutional layers mostly capture the main local features of images with 3 × 3 filters, and the last fully connected layer gives a binary classification according to the global feature connected from all local features. Unlike a plain convolutional neural network (CNN), ResNet avoids gradient vanishing by using identity and down-sampling blocks. The former keeps the shape of the input and output the same, while the latter halves the size of the output and doubles the number of channels. By adding direct paths, information of the input or gradient is allowed to pass through multiple layers to improve accuracy. U-Net32 consists of four down-sampling and four up-sampling steps, which reduce the 512 × 512 × 1 input image to a 32 × 32 × 256 representation, which is then up-sampled to a 512 × 512 × 2 output. During down sampling, each step contains a convolution block, followed by a max pooling layer and a dropout layer. Up sampling has each step consisting of a transpose convolution layer, followed by a dropout layer and a convolution block. A key feature of the U-Net architecture is that the convolutional kernel output from the encoding half of the network is concatenated with each corresponding decoding step, which helps preserve the details of the original image. The final layer consists of a convolution with two 1 × 1 kernels, which outputs a score for each of two classes: belonging to the pancreas or not. The final segmentation is achieved by selecting the class with the highest score for each pixel. We accelerated the training process by using z-score normalization and batch normalization layers in all sub-networks. At the same time, a dropout layer and L2 regularization are used to prevent overfitting.

All training, validation, and testing processes were performed in TensorFlow on a NVIDIA GeForce GTX 1050 Ti GPU. Adam optimizer 19 was used with default parameters β1 = 0.9 and β2 = 0.999. The dropout rate of the dropout layer was 0.4. Loss was calculated according to cross entropy. ResNet18's batch size was set to 32, and the epoch to 100. Learning rate was initialized at 1 × 10^-3^, and reduced by a factor of 10 every 20 epochs. U-Net32 had a batch size of 2 and epoch of 50. Learning rate was initialized at 5 × 10^-4^, and reduced by a factor of 20 every 20 epochs. ResNet34 had a batch size of 32, epoch of 86, and learning rate of 5 × 10^-5^.

### Statistical analysis

The FEE-DL model's performance was evaluated using accuracy (Acc) and F1 score (F1). The accuracy is defined as:

Acc = (TP + TN) / (TP + FP + TN + FN) (1);

where TP, TN, FP, and FN are the numbers of true positive, true negative, false positive, and false negative detections, respectively. F1 score is the harmonic average of precision and recall:

Precision = TP / (TP + FP) (2)

Recall = TP / (TP + FN) (3)

F1 = 2 × Precision × Recall/(Precision + Recall) (4)

The evaluation of segmentation considered two metrics: dice similarity coefficient (DSC) 20 and mean intersection over union (MIoU) 21. They are slightly different measures of the similarity between the ground truth and algorithm's prediction.

The DSC is defined as:

DSC = 2 × TP / (FP + 2 × TP + FN) (5);

and MIoU is the average of the intersection over union (IoU) in each category:

IoU = TP / (FP + TP + FN) (6)

MIoU = (∑*_n_* IoU) / *n* (7)

The number of categories, *n*, is two here, representing whether or not the pixel belongs to the pancreas area.

A receiver operating characteristic (ROC) curve is applied to visualize the performance of the final model, with its X and Y axes defined as the rates of false (FPR) and true (TPR) positives, respectively. In addition, the area under the ROC curve (AUC) is a classic quantitative metric. The closer the ROC curve is to the upper left corner, the higher the AUC value, and the more satisfactory the model's performance.

### Saliency map

A saliency map 22 improves the reliability of the model's diagnoses by computing the derivative of the correct class scores with respect to the image pixels, therefore highlighting the areas contributing most to the neural network's decision. As a visualization analysis tool, it can assist radiologists' understanding of the computer-aided decision and so enhance the model's credibility.

## Results

### Performance of sub-networks

**Figure 5:**
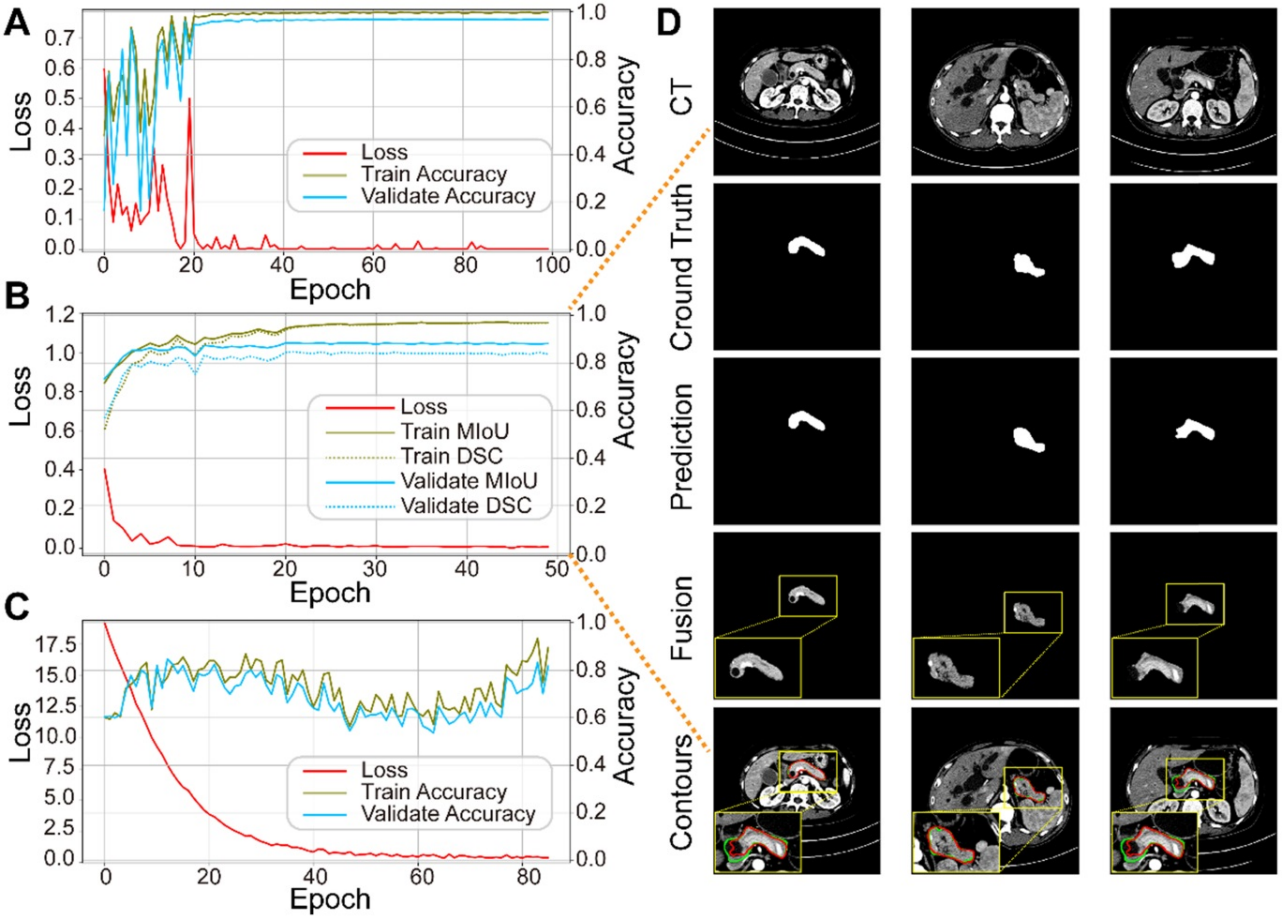
Performance of each sub-network in the training and validation datasets. **(A)** ResNet18 for pancreas location. **(B)** U-Net32 for pancreas segmentation. **(C)** ResNet34 for pancreatic tumor diagnoses. **(D)** Representative results of pancreas segmentation. Rows from top to bottom are input CT images, ground truth, prediction, fusion results, and pancreas contours in CT, respectively, where radiologists' annotations are shown in green and computerized segmentation is displayed in red. Higher resolution images are also shown on the lower left side.

We trained the three sub-networks independently, and ultimately tested the whole model. **Figure 5A-C** plots the loss and accuracy for the training and validation datasets**,** and **Table 3** lists the corresponding values. Valid images after data augmentation were randomly divided into three groups at a ratio of 6:2:2 for training, validating, and testing, respectively, each sub-network. When neither accuracy nor loss improved further, training process was stopped. **Figure 5D** displays three representative segmentation results from the training dataset, showing the pancreas, as a typical small organ, occupying only a very small fraction (~1.3%) of each CT image. Comparison of the prediction and ground truth reveals good performance in segmenting pancreas images of different shapes and sizes. Image fusion obtained more abundant pancreas information for diagnosis. The DSC and MIoU quantitatively describe the degree of overlap between the labeled (green contours) and predicted (red contours) results. The MIoU values of the three examples from left to right are 98.1%, 95.3%, and 88.2%, and the DSC values are 98.1%, 95.1%, and 87.0%, respectively.

### Performance of the FEE-DL model

**Figure 6:**
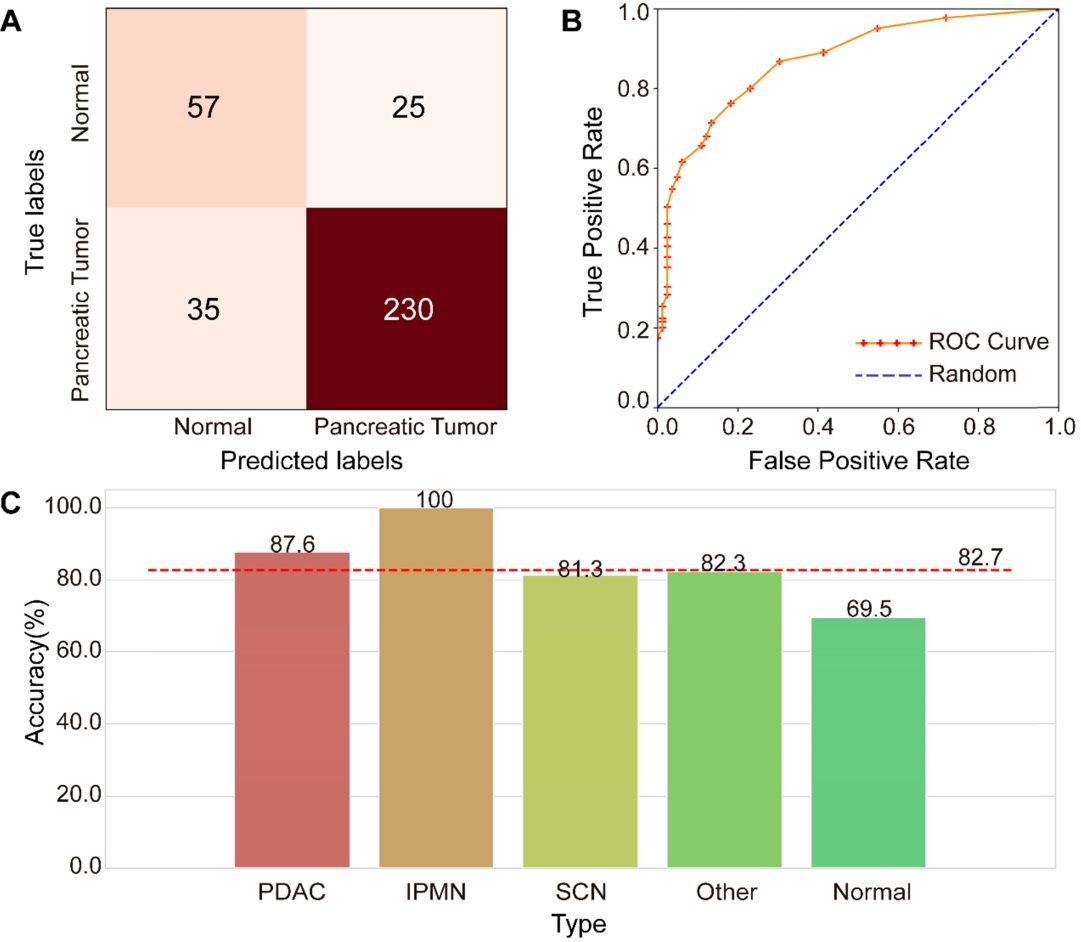
Performance of the FEE-DL model. **(A)** Confusion matrix. **(B)** Receiver operating characteristic (ROC) curves of the model and random prediction for comparison. The area under the curve (AUC) was 0.871. **(C)** Prediction accuracy of different pancreatic tumors with respect to the average accuracy (82.7%). (IPMN, intraductal papillary mucinous neoplasm; PDAC, pancreatic ductal adenocarcinoma; SCN, serous cystic neoplasm).

An independent testing dataset of 107,036 images from 265 tumor-positive examples and 82 negative controls was used to evaluate the proposed model. Only 62,649 images remained after image screening, which means that only 58.5% of the original clinical CT images in the testing dataset were used for deep-learning diagnosis, indicating the importance of image screening. The confusion matrix shown in **Figure 6A** represents the model's performance in detecting pancreatic tumors. The confusion matrix shows that it achieved an accuracy of 82.7% (287/347) and F1 score of 88.5%. The specificity is 69.5% (57/82), and the sensitive is 86.8% (230/265), indicating a strong performance in tumor detection. The ROC analysis in **Figure 6B** demonstrates the model's validity for prediction, with an AUC of 0.871, which compares favorably with random prediction (AUC = 0.5). We further explored the accuracy of the FEE-DL model in diagnosing whether there is a tumor for patients with each type of pancreatic tumor in the testing dataset, and compared them with the average accuracy, as shown in **Figure 6C**. The results indicate that PDAC and IPMN are more accurately identified than the average accuracy for all tumors, indicating the model's greater suitability for detecting them specifically. The low accuracy of identifying normal cases may be caused by the imbalance of normal and pancreatic tumor cases in the training dataset. Note that although there is no SCN case in the training dataset, the model can still predict its occurrence, demonstrating the model's robustness. The average testing time per patient was about 18.6 s from inputting the original abdominal CT image to diagnosis. These results indicate that the proposed model can rapidly diagnose pancreatic tumor with high accuracy.

**Figure 7:**
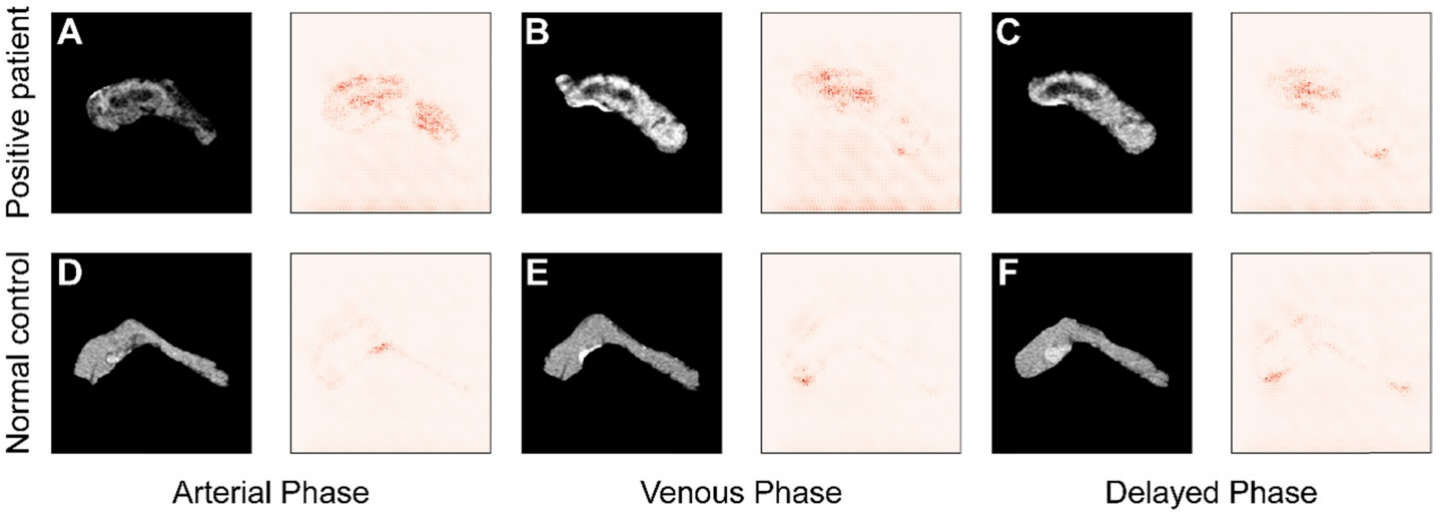
Comparison of saliency maps for **(A-C)** a tumor patient and **(D-F)** a normal control in different angiography phases: left, arterial phase; center, venous phase; and right, delayed phase.

To improve the interpretability of the diagnoses, the saliency maps in **Figure 7** visualize the regions that most influence the model's decision in the testing images. Darker pixels, representing those with more influence, are the basis for diagnosis. **Figure 7A-C** shows the results from a patient with pancreatic tumor, and in contrast **Figure 7D-F** represents results from a normal case in arterial, venous, and delayed phase, respectively. Comparing the saliency maps in **Figure 7A** and **D** shows that in the tumor case pancreatic duct dilation and heterogeneous density are emphasized, corresponding to the lesions in the CT image. In contrast, the saliency map of a normal case has no area obviously highlighted. Similar applies to the comparisons of **Figure 7B** and **E** and **Figure 7C** and **F**. These results show that the model focuses most on the tumor, and extracts rich information from that region, indicating that its diagnostic basis is similar to that of clinicians.

## Discussion

AI can play an important role in the diagnosis and treatment of disease, health management, drug research and development, and precision medicine. Its application to observing the pancreas has been hindered by the pancreas being highly variable in shape, size, and location, while occupying only a very small fraction of a CT image. The result has been low accuracy and poor diagnostic efficiency. This study proposes a FEE-DL model to assist pancreatic tumor diagnosis from original abdominal CT images without any manual preprocessing. The model includes four steps: image screening, pancreas location, pancreas segmentation, and pancreatic tumor diagnosis; and achieves an AUC of 0.871, F1 score of 88.5%, and accuracy of 82.7% on an independent testing dataset of 347 patients. A further advantage of this work is the establishment of a larger dataset containing more pancreatic tumor types (PDAC, IPMN, SCN, and other) than previously available. This can assist the deep-learning system to identify images of various types of pancreatic tumor. The model independently identifies different pancreatic tumors, and performs strongest in detecting IPMN and PDAC. Another strength is the end-to-end automatic diagnosis, which takes only about 18.6 s per patient from inputting the original abdominal CT image to a diagnosis result. It can handle and meaningfully process massive sets of data quickly, accurately, and inexpensively, making it suitable for clinical use with important potential for diagnosing and recommending treatments. For example, the model could be used for large-scale pre-diagnosis during physical examination, or to assist diagnosis at low-level hospitals with scarce resources. A final feature of the model that can help improve its reliability is its ability to produce saliency maps to identify the areas of greatest importance to its diagnostic decision making. While our method uses only evidence from CT images, clinicians have an access to additional data such as patients' health records and testimony, and definite diagnoses and treatment plans should continue to be based on the clinical judgment of specialists and not solely on the results of a deep-learning system.

In addition to classifying pancreatic tumors, assessing the tumor stage, following up after treatment to assess response, and predicting the resectability of a given pancreatic tumor to guide surgery are potentially important applications of our AI-based technique. Deep-learning algorithms have an important place in assisting clinical diagnosis. In the future, a multi-modal diagnostic model can be developed based on the fusion of different characteristics from CT images, MRI images and clinical examination data to further improve accuracy. In summary, this model can support clinical decision making by efficiently delivering accurate preoperative diagnoses that could aid the surgical management of pancreatic tumors.

## Supplementary Material

Supplementary tables.

## Figures and Tables

**Table 1:** Types of pancreatic tumor in the training and testing datasets

Type	Training dataset	Testing dataset
Normal	35	82
PDAC	209	170
IPMN	6	17
PNET	1	-
SCN	-	16
Other	68	62
All	319	347

IPMN, intraductal papillary mucinous neoplasms; PDAC, pancreatic ductal adenocarcinoma; PNET, pancreatic neuroendocrine tumors; SCN, serous cystic neoplasms.

**Table 2:** Patient characteristics in the training and testing datasets

Number	Training dataset	Testing dataset
Patients	319	347
Mean age	63.3 (Range: 37-90)	61.8 (Range: 24-88)
Male	211 (~66.1%)	216 (~62.2%)
Female	108 (~33.9%)	131 (~37.8%)
Abnormal images	133,591 (284 people)	90,194 (265 people)
Normal images	10,354 (35 people)	16,842 (82 people)
Total images	143,945	107,036

**Table 3:** Performance of each sub-network

Sub-network	Epoch	Training Acc (%)	Validation Acc (%)	Testing Acc (%)	Training Loss
ResNet18	100	99.8	96.5	97.1	5.81e^-7^
U-Net32	50	96.9 (MIoU)	88.2 (MIoU)	88.0 (MIoU)	1.54e^-3^
		96.8 (DSC)	83.7 (DSC)	83.7 (DSC)	
ResNet34	86	89.5	81.7	82.2	0.395

Acc, accuracy; DSC, dice similarity coefficient; MIoU, mean intersection over union.

## Abbreviations

AI: artificial intelligence
AUC: area under the curve
CNN: convolutional neural network
CT: computed tomography
DSC: dice similarity coefficient
FEE-DL: fully end-to-end deep-learning
FPR: false positive rate
IoU: intersection over union
IPMN: intraductal papillary mucinous neoplasms
MIoU: mean intersection over union
PDAC: pancreatic ductal adenocarcinoma
PNET: pancreatic neuroendocrine tumors
ROC: receiver operating characteristic
ROI: regions of interest
SCN: serous cystic neoplasms
TPR: true positive rate
