# Supplementary material for: Fully end-to-end deep-learning-based diagnosis of pancreatic tumors
Source: Theranostics. 2021 Jan 1;11(4):1982–90. doi: 10.7150/thno.52508 (PMC7778580; doi:10.7150/thno.52508)
Supplement: Supplementary file 1 — Supplementary tables. [file thnov11p1982s1.pdf]

Table S1. Detailed architecture of ResNet18.

				ResNet18			
Layer	Kernel Size	Kernel Number	Kernel Stride	DS_Kernel Size	DS_Kernel Number	DS_Kernel Stride	FC Units
Conv	7*7	64	2	-	-	-	-
MaxPool	3*3	-	2	-	-	-	-
ID Block*2	3*3	64	1	-	-	-	-
DS Block	3*3	128	1	3*3	128	2	-
ID Block	3*3	128	1	-	-	-	-
DS Block	3*3	256	1	3*3	256	2	
ID Block	3*3	256	1	-	-	-	-
DS Block	3*3	512	1	3*3	512	2	
ID Block	3*3	512	1	-	-	-	-
AvgPool	7*7	-	1	-	-	-	-
FC	-	-	-	-	-	-	512

Table S2. Detailed architecture of U-Net32.

Layer	U-Net32 Kernel Size	Kernel Number	Kernel Stride
Conv Block	3*3	16	1
MaxPool	2*2	-	2
Conv Block	3*3	32	1
MaxPool	2*2	-	2
Conv Block	3*3	64	1
MaxPool	2*2	-	2
Conv Block	3*3	128	1
MaxPool	2*2	-	2
Conv Block	3*3	256	1
Trans Conv	2*2	128	2
Conv Block	3*3	128	1
Trans Conv	2*2	64	2
Conv Block	3*3	64	1
Trans Conv	2*2	32	2
Conv Block	3*3	32	1
Trans Conv	2*2	16	2
Conv Block	3*3	16	1
Conv	1*1	2	1

Table S3. Detailed architecture of ResNet34.

	ResNet34						
Layer	Kernel Size	Kernel Number	Kernel Stride	DS_Kernel Size	DS_Kernel Number	DS_Kernel Stride	FC Units
Conv	7*7	64	2	-	-	-	-
MaxPool	3*3	-	2	-	-	-	-
ID Block*3	3*3	64	1	-	-	-	-
DS Block	3*3	128	1	3*3	128	2	-
ID Block*3	3*3	128	1	-	-	-	-
DS Block	3*3	256	1	3*3	256	2	
ID Block*5	3*3	256	1	-	-	-	-
DS Block	3*3	512	1	3*3	512	2	
ID Block*2	3*3	512	1	-	-	-	-
AvgPool	7*7	-	1	-	-	-	-
FC	-	-	-	-	-	-	512

AvgPool, average-pooling; BN, batch normalization; Conv, convolution; DS, down sampling; FC, fully connected; ID, identity; MaxPool, max-pooling; ReLU, rectified linear unit; Trans, transposed.
